# Comparisons of the risk of myopericarditis between COVID-19 patients and individuals receiving COVID-19 vaccines: a population-based study

**DOI:** 10.1007/s00392-022-02007-0

**Published:** 2022-03-25

**Authors:** Oscar Hou In Chou, Jiandong Zhou, Teddy Tai Loy Lee, Thompson Kot, Sharen Lee, Abraham Ka Chung Wai, Wing Tak Wong, Qingpeng Zhang, Shuk Han Cheng, Tong Liu, Vassilios S. Vassiliou, Bernard Man Yung Cheung, Gary Tse

**Affiliations:** 1 Division of Clinical Pharmacology, Department of Medicine, School of Clinical Medicine, University of Hong Kong, Hong Kong, China; 2 Epidemiology Research Unit, Cardiovascular Analytics Group, Hong Kong, China; 3 Nuffield Department of Medicine, University of Oxford, Oxford, UK; 4 Department of Emergency Medicine, School of Clinical Medicine, University of Hong Kong, Hong Kong, China; 5 Department of Anaesthesia, Princess Margaret Hospital, Hong Kong, China; 6 School of Life Sciences, Chinese University of Hong Kong, Hong Kong, China; 7 School of Data Science, City University of Hong Kong, Hong Kong, China; 8 Department of Infectious Diseases and Public Health, City University of Hong Kong, Hong Kong, China; 9 Tianjin Key Laboratory of Ionic-Molecular Function of Cardiovascular Disease, Department of Cardiology, Tianjin Institute of Cardiology, Second Hospital of Tianjin Medical University, Tianjin, 300211 China; 10 Department of Cardiology, Norwich Medical School, University of East Anglia, Norfolk, UK; 11 Kent and Medway Medical School, Canterbury, Kent UK; 12 Norwich and Norwich University Hospital, Colney Lane, Norwich, NR4 7UQ UK; 13 Department of Medicine, Honorary Consultant, Queen Mary Hospital, Hong Kong, China

**Keywords:** COVID-19, Vaccine, Myopericarditis, Myocarditis, Pericarditis

## Abstract

**Background:**

Both COVID-19 infection and COVID-19 vaccines have been associated with the development of myopericarditis. The objective of this study is to (1) analyse the rates of myopericarditis after COVID-19 infection and COVID-19 vaccination in Hong Kong, (2) compared to the background rates, and (3) compare the rates of myopericarditis after COVID-19 vaccination to those reported in other countries.

**Methods:**

This was a population-based cohort study from Hong Kong, China. Patients with positive RT-PCR test for COVID-19 between 1st January 2020 and 30th June 2021 or individuals who received COVID-19 vaccination until 31st August were included. The main exposures were COVID-19 positivity or COVID-19 vaccination. The primary outcome was myopericarditis.

**Results:**

This study included 11,441 COVID-19 patients from Hong Kong, four of whom suffered from myopericarditis (rate per million: 326; 95% confidence interval [CI] 127–838). The rate was higher than the pre-COVID-19 background rate in 2019 (rate per million: 5.5, 95% CI 4.1–7.4) with a rate ratio of 55.0 (95% CI 21.4–141). Compared to the background rate, the rate of myopericarditis among vaccinated subjects in Hong Kong was similar (rate per million: 5.5; 95% CI 4.1–7.4) with a rate ratio of 0.93 (95% CI 0.69–1.26). The rates of myocarditis after vaccination in Hong Kong were comparable to those vaccinated in the United States, Israel, and the United Kingdom.

**Conclusions:**

COVID-19 infection was associated with significantly higher rate of myopericarditis compared to the vaccine-associated myopericarditis.

**Graphical abstract:**

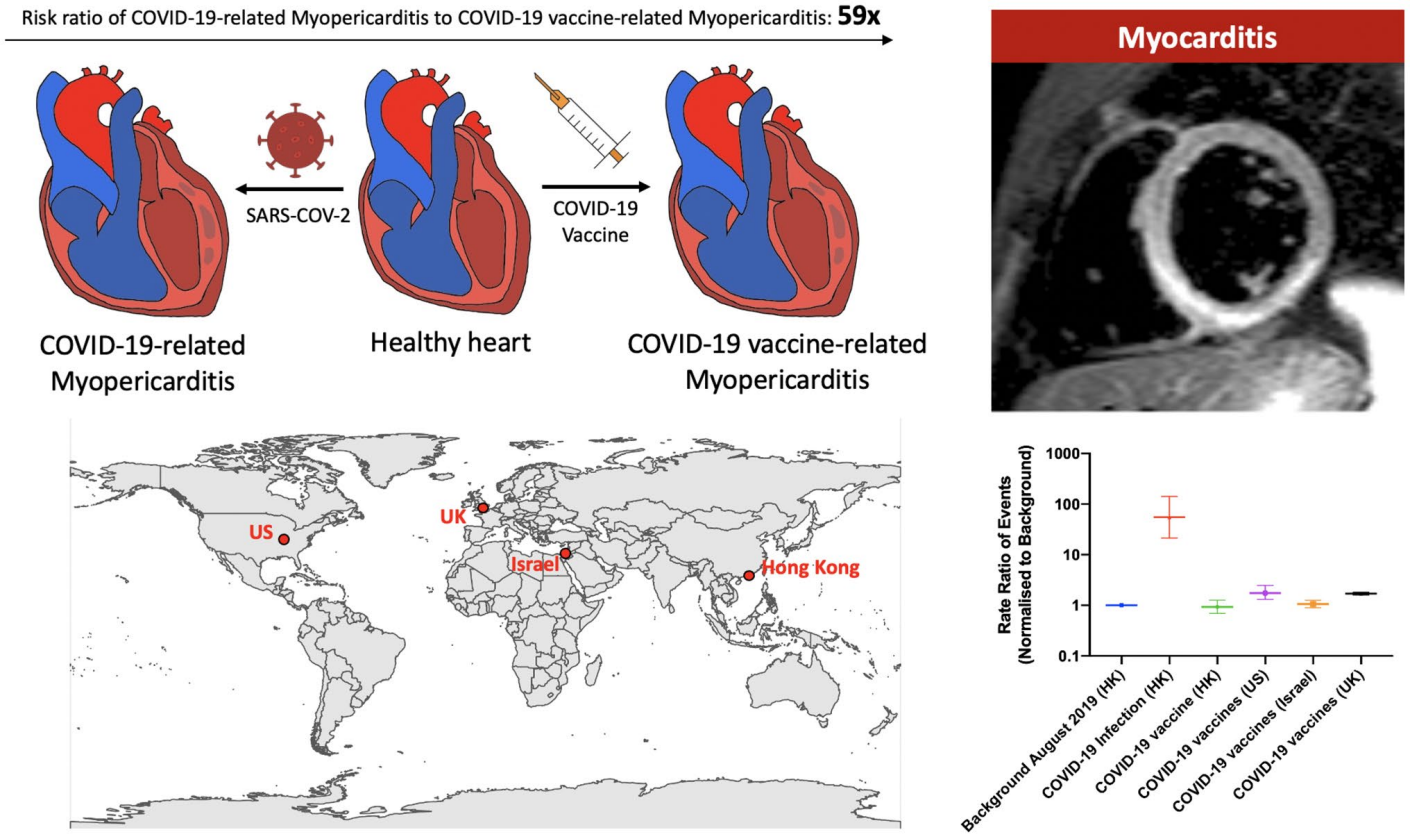

**Supplementary Information:**

The online version contains supplementary material available at 10.1007/s00392-022-02007-0.

## Introduction

Since the beginning of the COVID-19 pandemic, cases of myocarditis and pericarditis related to the infection have been described [1, 2]. Recent studies have also reported possible associations between COVID-19 vaccines and the risk of myo-pericardial inflammation, raising concerns about vaccination uptake especially in teenagers. Previously, a preprint study published went viral as it miscalculated the denominator, such that that 1 in 1000 would develop heart inflammation upon COVID-19 vaccination. In this study, we conducted a population-based study using data from Hong Kong, China to determine the rates of myopericarditis after COVID-19 infection and COVID-19 vaccination, comparing them to background rates and addressing the rate ratio of vaccine versus COVID-19 myopericarditis.

## Methods

This population-based retrospective cohort study was approved by the Institutional Review Board of the University of Hong Kong/Hospital Authority Hong Kong West Cluster (UW 20–250). The need for informed consent was waived by the Ethics Committee owing to its observational retrospective nature. Patients who tested positive for COVID-19 by real-time polymerase chain reaction (RT-PCR) at any of the Hong Kong public hospitals or outpatient clinics between 1st January 2020 and 30th June 2021 were included. The background number of myopericarditis in 2018 and 2019 in Hong Kong were obtained using the ICD codes 420.9, 422.x, 423.9, and 429.0 suggested by a previously published paper [3]. The data were obtained from the local electronic healthcare database, Clinical Data Analysis and Reporting System as reported previously [4]. The primary outcome was myopericarditis. The vaccination data of different countries were extracted using keywords labelled “Myocarditis” and “Pericarditis” upon searching PubMed and the official reports of Hong Kong and the United Kingdom. The data in Hong Kong and the United Kingdom were up to 31st August 2021 and 29th September 2021, respectively.

The rates after COVID-19 infection were calculated by dividing the number of myocarditis and pericarditis patients by the number of RT-PCR positive COVID-19 patients and adjusted to 14 days. The number of doses was defined as the sum of the number of people who received the first and second doses. The hybrid Wilson/Brown method was used to calculate 95% confidence intervals for the rates (Supplementary Table 1). The analysis was conducted using PRISM (Version: 9.0.0) and RStudio (Version: 1.4.1103).

## Results

**Fig. 1:**
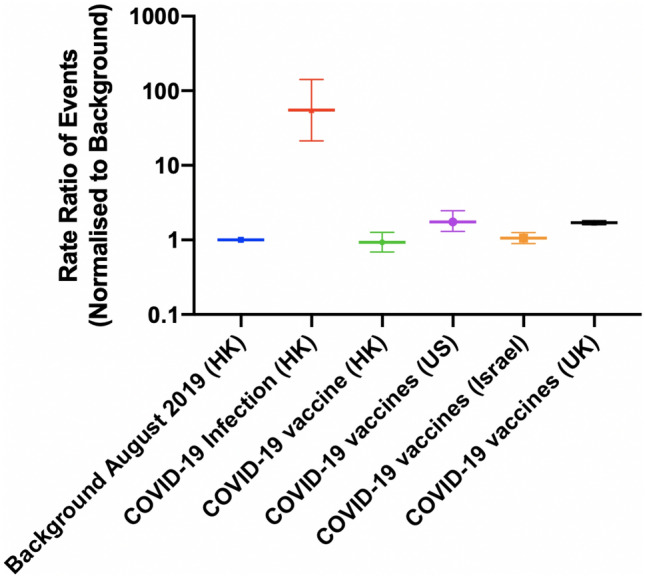
The rate ratio of the events after COVID-19 infection and COVID-19 vaccination

A total of 11,441 COVID-19 patients from Hong Kong were included, of which four (mean age: 71.2; 50% male) suffered from myopericarditis (rate per million: 326; 95% confidence interval [CI] 127–838). The rate was higher than the pre-pandemic background rate in 2019 (rate per million: 5.9, 95% CI 5.4–6.5) with a rate ratio of 55.0 (95% CI 21.4–141.; Table 1). As of 31st August 2021, 2,811,500 doses of CoronaVac (37.05%) and 4,776,700 doses of BioNTech (62.95%) have been administered [5]. The mean age of the individuals vaccinated with CoronaVac was 61.58 (SD: 11.08) and with Comirnaty was 56.81 (SD: 13.43) [6]. The proportion of male in individuals vaccinated with CoronaVac was 48.7% and with BioNTech was 47.4% [6]. 42 patients developed myopericarditis within 14 days after vaccination, 16 of which belonged to the 12–15 age group. Out of the 42 patients, 41 patients were vaccinated with BioNTech vaccine, while one patient was vaccinated with CoronaVac vaccine. Compared to the background rates, the rate of myopericarditis among the vaccinated subjects in Hong Kong was similar (rate per million: 5.5; 95% CI 4.1–7.4) with a rate ratio of 0.93 (95% CI 0.69–1.26). The rates of myocarditis after vaccination in Hong Kong were comparable to those vaccinated in the United States, Israel, and the United Kingdom (Fig. 1).

**Table 1:** Incidence rate and rate ratio of myocarditis and pericarditis after COVID-19 infection and vaccination

Outcomes	Background 2019 (Hong Kong, China)	Covid-19 infection (Hong Kong, China)	COVID-19 vaccine (Hong Kong, China)* [5]	COVID-19 vaccines (US) [12]	COVID-19 vaccines (Israel) [2]	COVID-19 vaccines (UK) [13]**
Diagnosis	Myopericarditis	Myopericarditis	Myopericarditis	Myopericarditis	Myocarditis	Myopericarditis
Type of vaccines	NA	NA	CoronaVac BNT162b2	BNT162b2 mRNA-1273 Ad26.COV2.S	BNT162b2	BNT162b2 mRNA-1273 ChAdOx1
Cases	529	4	42	57	136	947
Unit	Persons	Persons	Doses	Doses	Doses	Doses
Time interval	183 days	15 days	≤ 14 days	3.5 days (myocarditis) 20 days (pericarditis)	≤ 21 days (1^st^ dose) ≤ 30 days (2^nd^ dose)	NA
Persons or doses received	6,819,672	11,441	7,588,200	3,530,507	10,568,331	93,600,000
Rate per million persons or doses per 14 days (95% CI)	5.9 (5.4, 6.5)	326 (127, 838)	5.5 (4.1, 7.4)	10.3 (7.7, 14.7)	6.27 (5.3, 7.4)	10.1 9.5, 10.8
Rate ratio (95% CI)	1 (baseline)	55.0 (21.4, 141)	0.93 (0.69 1.26)	1.74 (1.30, 2.47)	1.06 (0.89, 1.25)	1.70 (1.60, 1.82)

*Cases for ≥ 12 years old only

**The rate and rate ratio of the COVID-19 vaccines (UK) were not adjusted to 14 days as the time period was not available

**Fig. 2:**
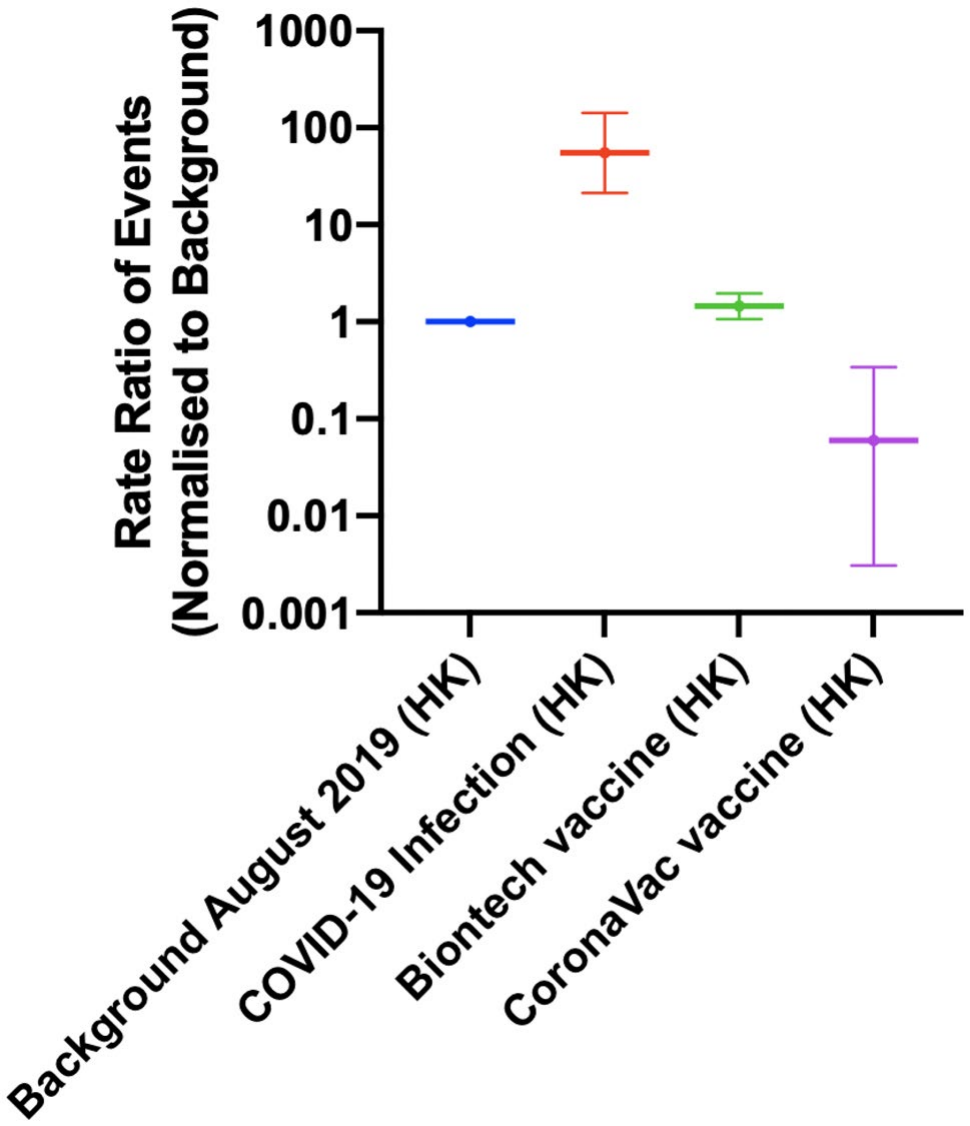
The rate ratio of the events after COVID-19 infection and COVID-19 vaccination in Hong Kong stratified by vaccine type

**Fig. 3:**
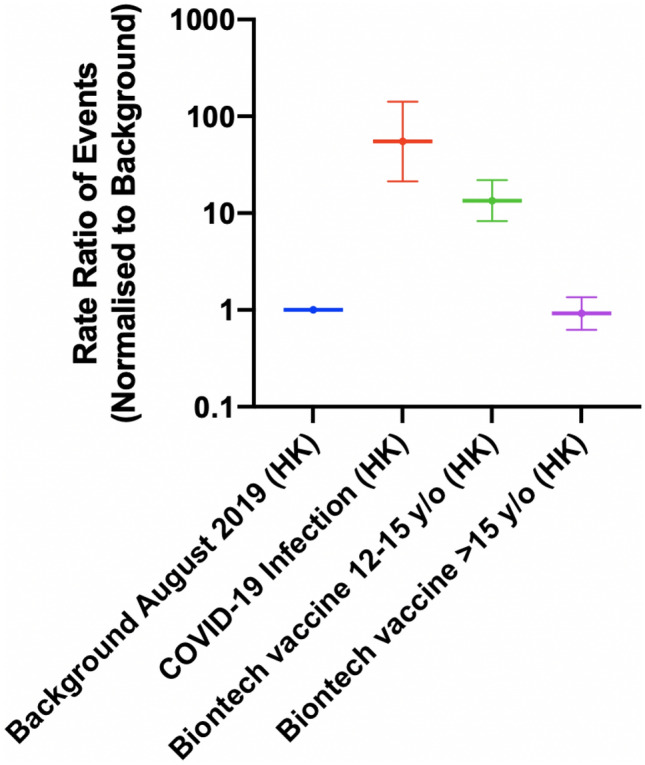
The rate ratio of the events after COVID-19 infection and BioNTech vaccination in Hong Kong stratified by age

**Fig. 4:**
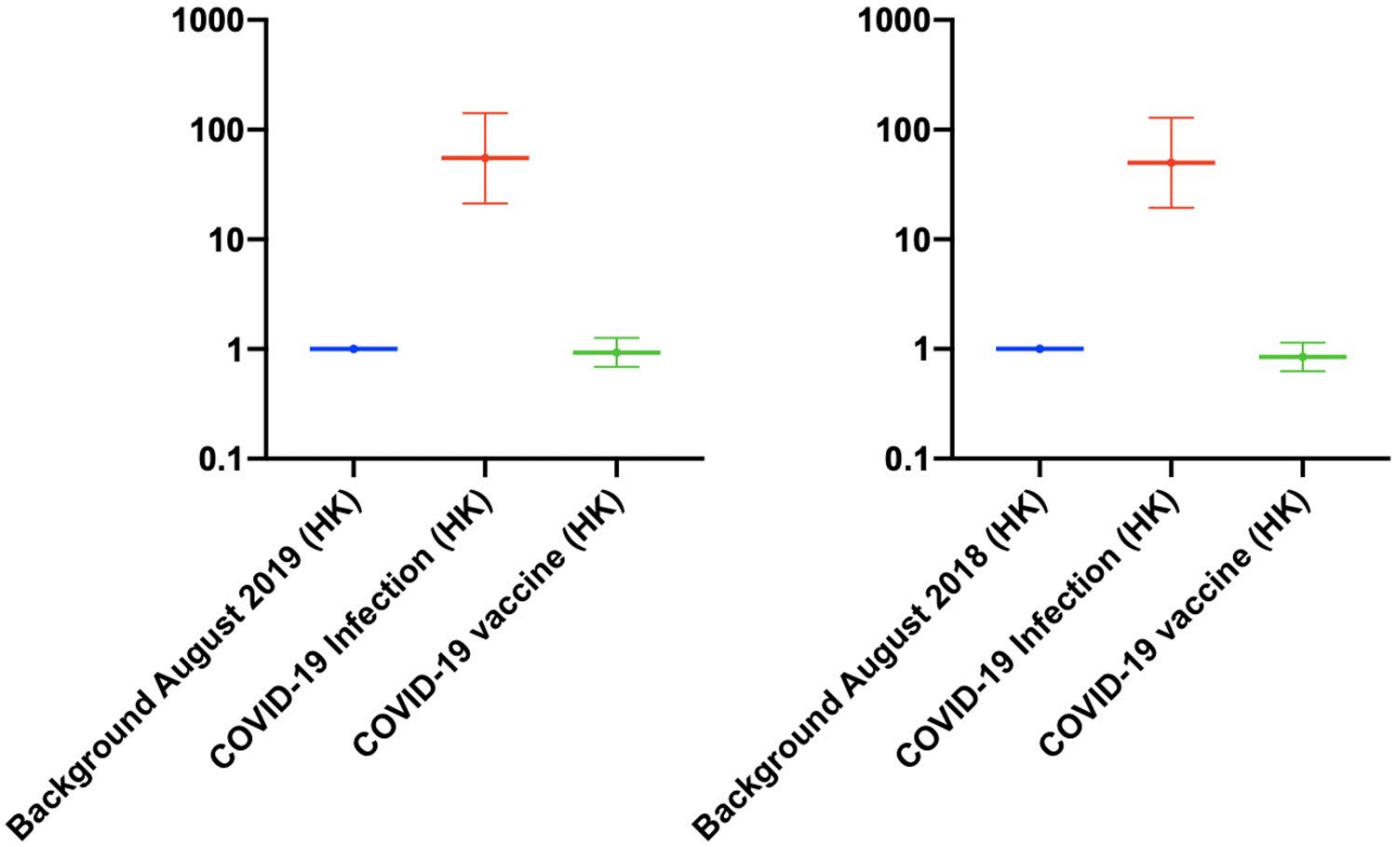
Sensitivity testing 1: the rate ratio of the events after COVID-19 infection and COVID-19 vaccination in Hong Kong with different background years [5]. The background period was between March and August in 2018–2019

The rate ratio of the myopericarditis were stratified according to the vaccine types (Fig. 2, Supplementary Table 2). Compared to the background, the rates of myopericarditis were higher among individuals vaccinated with BioNTech (Rate ratio: 1.45; 95% CI 1.07, 1.96) and lower among those vaccinated with CoronaVac vaccine (rate ratio: 0.06; 95% CI 0.003, 0.34). Among the individuals vaccinated with BioNTech, the rate of myopericarditis was similar to the background among individuals aged > 15 years (rate ratio: 0.92; 95% CI: 0.62, 1.36). Meanwhile, for individuals aged 12–15 years, the rate was higher than the background (Rate ratio: 13.5; 95% CI 8.30, 21.9) (Fig. 3, Supplementary Table 3). Sensitivity analysis was conducted to verify the trend of our data. The trend of myopericarditis after COVID-19 infection and vaccination in Hong Kong remained consistent while comparing the background to March-August in 2018 (Fig. 4).

## Discussion

The main finding from our study is that COVID-19 patients had a higher rate of myopericarditis compared to the pre-COVID-19 era background. With COVID-19 vaccination, the rate of myopericarditis was significantly lower than the COVID-19 positive patients.

Our team recently developed a predictive model to identify COVID-19 patients at risk of severe disease [7]. Using an updated dataset, we found that 0.035% of the COVID-19-infected patients developed myopericarditis. This is significantly higher than the baseline, reflecting the association between COVID-19 infection and myopericarditis. Previously, it was described that among 718,365 patients with COVID-19, approximately 6.5% developed new-onset myopericarditis [8]. This higher rate might be an overestimate, since most of the patients presented with mild COVID-19 symptoms may not get admitted and diagnosed, thus, not registered with the electronic medical records. Our cases represented population-based data locally in Hong Kong, which has practised meticulous contact tracing since the pandemic; the number of total COVID-19 patients in our cohort would, therefore, likely reflect most of the infected patients in the community [9].

The link between COVID-19 vaccination and myopericarditis was raised in Israel in May 2021. The results demonstrated that COVID-19 vaccinations are associated with a lower myopericarditis rate than the COVID-19-infected patients. This indicated that the COVID-19 vaccination might protect the vaccinated people from the myocardial injury caused by SARS-CoV-2 infection [10]. The differences in the outcome suggested that the myopericarditis upon vaccination is unlikely due to the mimicry between the spike proteins [11]. While the rate of myopericarditis was similar to the background in our data; this does not rule out the causation between the COVID-19 vaccine and myopericarditis. Indeed, the majority of myopericarditis developed after the second dose of the vaccine, in particular among the younger male (< 19 years old) [2]. While the rate of myopericarditis following COVID-19 infection is relatively low; further studies are needed to characterise the mechanism of myopericarditis after COVID-19 vaccination. Nonetheless, with the current increasing numbers in COVID-19 globally, it appears that every person will come in contact with SARS-CoV-2, and this is alarming given the overall rate of myopericarditis is much higher.

### Strengths and limitations

This study has several strengths. First, COVID-19 cases were identified by RT-PCR testing across the public sector. Therefore, missing cases are likely to be few. Second, possible cases of vaccine-related myopericarditis were reviewed by an expert panel, which examined the medical records independently. This adjudication has permitted the accurate classification of cases according to established international guidelines. Nevertheless, had we included possible cases rather than cases definitely linked to vaccinations, the rate ratios of myopericarditis cases in infected patients to cases occurring after COVID-19 vaccination would be even lower, and therefore does not alter our conclusion.

However, several limitations should be noted. First, the cohort included patients recruited from a single region and as such, is unable to account for any geographical heterogeneity that may exist. Second, the vaccination data reported in the local Department of Health did not provide the number of myopericarditis after the first and the second dose of vaccination, and therefore, the results were not stratified. Third, we might have missed COVID-19 myopericarditis in patients who might have died from acute heart failure complications due to myopericarditis but recorded as heart failure deaths. This, however, would have increased the difference between COVID-19 and vaccine-related myopericarditis, further strengthening the outcome of vaccination. Furthermore, the COVID-19-infected patients that were undetected by PCR were not included in this study. However, as Hong Kong has imposed a relatively strict policy in contact tracing and quarantine, we believe that this number is relatively low [9]. Finally, given the retrospective nature of this study, the incidence of myopericarditis can be underestimated.

## Conclusion

COVID-19 infection is associated with significantly higher rate of myopericarditis compared to the vaccine-associated myopericarditis.

## Supplementary Information

Below is the link to the electronic supplementary material.

Supplementary file1 (DOCX 22 KB)
