# Supplementary material for: Comparisons of the risk of myopericarditis between COVID-19 patients and individuals receiving COVID-19 vaccines: a population-based study
Source: Clin Res Cardiol. 2022 Mar 25;111(10):1098–103. doi: 10.1007/s00392-022-02007-0 (PMC8951670; doi:10.1007/s00392-022-02007-0)
Supplement: Supplementary file 1 — Supplementary file1 (DOCX 22 KB) [file 392_2022_2007_MOESM1_ESM.docx]

**Supplementary Appendix**

Step 1: Data extraction and calculations of event rates.

| **Myopericarditis (all)** | **COVID-19** | **COVID vaccine (both) Aug 2021** | **Background Hong Kong (August 2019)** | **Background Hong Kong (August 2018)** |
| --- | --- | --- | --- | --- |
| Cases | 4 | 42 | **529** | **578** |
| Persons or doses received | 11441  (Person) | 7588200 (dosage) | 6852459 (Person) | 6761194 (Person) |
| Rate | 0.00034962 | 5.53491E-06 | 7.75697E-05 | 8.54879E-05 |
| Rate per million | 349.6197885 | 5.534909465 | 77.56971154 | 85.48785872 |
| Negative | 11437 | 7588158 | 6819143 | 6760616 |

| **Myopericarditis (all)** | **COVID-19 vaccines (United States)** | **COVID-19 vaccine (Israel)** | **COVID-19 vaccines (United Kingdom)** |
| --- | --- | --- | --- |
| Cases | 57 | 136 | 947 |
| Persons or doses received | 3530507 | 10568331 | 93600000 |
| Rate | 1.61450E-05 | 1.28686E-05 | 1.01175E-05 |
| Rate per million | 16.14498994 | 12.86863555 | 10.11752137 |
| Negative | 3530450 | 10568195 | 93599053 |

Step 2: The hybrid Wilson/Brown method was used to calculate 95% confidence intervals for the rates.

| **Myopericarditis (all)** | **COVID-19** | **COVID vaccine (both) Aug 2021** | **Background Hong Kong (August 2019)** | **Background Hong Kong (August 2018) *** |
| --- | --- | --- | --- | --- |
| Mean | 0.00034962 | 5.53491E-06 | 5.93429E-06 | 6.54005E-06 |
| Upper Limit | 0.000898686 | 7.48097E-06 | 6.46197E-06 | 7.09537E-06 |
| Lower Limit | 0.000135968 | 4.09509E-06 | 5.4497E-06 | 6.02819E-06 |

* The background rate in Hong Kong (August 2019) was adjusted to 14 days

Step 3: Calculate the rate ratio with the background as the reference

| **Myopericarditis (all)** | **COVID-19** | **COVID vaccine (both) Aug 2021** | **Background Hong Kong (August 2019)** |
| --- | --- | --- | --- |
| Rate per million | 58.89454401 | 0.932693579 | 1 |
| Upper 95% CI | 151.3865217 | 1.260626078 |  |
| Lower 95% CI | 22.90430785 | 0.69006733 |  |

| **Myopericarditis (all)** | **COVID-19** | **COVID vaccine (both) Aug 2021** | **Background Hong Kong (August 2018)** |
| --- | --- | --- | --- |
| Rate per million | 53.45823536 | 0.846309341 | 1 |
| Upper 95% CI | 137.412647 | 1.143869379 |  |
| Lower 95% CI | 20.79010757 | 0.626154657 |  |

**Supplementary Table 1. The calculation of the rate ratio for the COVID-19 data in Hong Kong relative to the background in Hong Kong (August 2019).**

| **Outcomes** | **Background 2019**  **(Hong Kong, China)** | **Covid-19 infection**  **(Hong Kong, China)** | **BioNTech vaccine**  **(Hong Kong, China)[8]** | **CoronaVac vaccine**  **(Hong Kong, China)[8]** |
| --- | --- | --- | --- | --- |
| Diagnosis | Myopericarditis | Myopericarditis | Myopericarditis | Myopericarditis |
| Cases | 529 | 4 | 41 | 1 |
| Unit | Persons | Persons | Doses | Doses |
| Mean time interval | 183 days | 15 days | ≤14 days | ≤14 days |
| Persons or doses received | 6819672 | 11441 | 4776700 | 2811500 |
| Rate per million persons or doses per 14 days (95% CI) | 5.5  (4.1, 7.4) | 326  (127, 838) | 8.6  (6.3, 12) | 0.35  (0.35, 2.0) |
| Rate Ratio (95% CI) | 1 (baseline) | 55.0 (21.4, 141) | 1.45 (1.07, 1.96) | 0.06 (0.003, 0.34) |

**Supplementary Table 2. Incidence rate and rate ratio of myocarditis and pericarditis in Hong Kong after COVID-19 infection and vaccination stratified by vaccine available in Hong Kong.**

| **Outcomes** | **Background 2019**  **(Hong Kong, China)** | **Covid-19 infection**  **(Hong Kong, China)** | **BioNTech vaccine 12-15 y/o**  **(Hong Kong, China)[8]** | **BioNTech vaccine >15 y/o**  **(Hong Kong, China)[8]** |
| --- | --- | --- | --- | --- |
| Diagnosis | Myopericarditis | Myopericarditis | Myopericarditis | Myopericarditis |
| Cases | 529 | 4 | 16 | 26 |
| Unit | Persons | Persons | Doses | Doses |
| Mean time interval | 183 days | 15 days | ≤14 days | ≤14 days |
| Persons or doses received | 6819672 | 11441 | 200000 | 4576700 |
| Rate per million persons or doses per 14 days (95% CI) | 5.5  (4.1, 7.4) | 326  (127, 838) | 80  (49, 130) | 5.5  (3.7, 8.1) |
| Rate Ratio (95% CI) | 1 (baseline) | 55.0 (21.4, 141) | 13.5 (8.30, 21.9) | 0.92 (0.62, 1.36) |

**Supplementary Table 3. Incidence rate and rate ratio of myocarditis and pericarditis in Hong Kong after COVID-19 infection and BioNTech vaccination stratified by age in Hong Kong.**
